# Distension of Gall Bladder: Operation

**Published:** 1892-02

**Authors:** J. Cummings

**Affiliations:** Austin


For Daniel's Texas Medical Journal.

### REPORT OF A CASE OF DISTENSION OF THE GALL BLADDER.

BY J. CUMMINGS, M. D., AUSTIN.

Read at Austin District Medical Association December 17, 1891.

WAS called, June 10th, 1891, to see B. F. C. White, age fifty-five. Found him suffering with fever, and intense tenderness over the abdomen, especially on the right side, in the region of and below the liver. The skin was not jaundiced, but presented a dull, dusky color. Had been complaining several months of stomach; so much so that two physicians had suspected cancer of this organ.

Dullness extended below the ribs on the right side, and simulated in this respect—inflamation in the region of the cæcum. A pretty brisk purgative and anodyne was prescribed, with only temporary relief. His suffering became so great by the 14th, that I requested Dr. M. A. Taylor to assist me in an exploratory operation. After cutting through the peritoneum, the finger could be passed above the induration, in the region of the stomach, but nothing except a hardened mass below could be detected that appeared abnormal. Two threads were passed (after an exploratory puncture with an aspiraotr needle, showing the character of the contents), before incising the gall bladder, by tension on the threads, the gall bladder could be held firmly against the abdominal wall, when a free opening was made with a bistury, and a large quantity of pure bile escaped, probably not less than one pint. Dr. Taylor and I explored the inside of the gall cyst—using the index finger of the right hand, but could detect no gall stones or other hardness, or any condition pointing to disease, other than the obstruction, evident in the cystic duct, leading to the accumulation of bile. On the 16th less bile discharged than on the 15th, on which date it was very profuse, saturating the bed and all cloths used for protecting his person. From the amount discharged, I must think the estimate of near three pounds of bile, secreted in twenty-four hours in the healthy man, as given in our works on physiology, to be about right. On the 17th, 18th and 19th, no bile discharged. On the 20th bile began again to flow. On the 22nd, still running freely. Extracted all stitches except threads in gall bladder. On the 22nd still free discharge of bile, patient was allowed to get up. July 6th discharge of bile had stopped several days. Dr. J. E. Cummings, of Uvalde, visited the patient with me. July 9th was called at night: patient was suffering with severe pain and cramps. Excessive tenderness over the abdomen. On the 10th probed wound without effecting flow of bile—some discharge, but not characteristic. July 11, soreness somewhat reduced, but redistension of the gall cyst more apparent; used probe again without result, the opening evidently being too small to allow exit of the thick bile. I was so confident of the distension of the gall bladder, and urged by a weak, short pulse, and impending disolution without relief, I inserted a large trocar and canula into the cyst, with the gratifying result of relieving the accumulation, and found that some pus discharged with the bile, also some small gall stones, not larger than a pea each, two or three in number. On the 13th, bile was flowing profusely, improvement, however, slower than after first operation. After a number of days he showed the decided benefit derived from the treatment. After this misfortune, we kept the wound open with first drainage tube, and later, rubber tube, devised for the purpose. Improvement has been constant since, and he has gained his usual flesh, and he is now apparently well; there being complete closure of the external opening. At first the stools were pale, without bile, but later became natural, and remained so.

In the London *Lancet*, Mr. Arbuthnot Lane reports a case where a boy received an injury, followed by distension of the gall bladder, and suspected partial rupture of ducts, with evidence of bile having been poured into abdominal cavity, without serious symptoms. In my case, however, when I suspected some discharge of bile into abdominal cavity, the symptoms grew alarming, as shown by soreness and swelling of the abdomen, with weak, short pulse, only relieved by re-establishing the external outlet for the bile.

In a recent number of the *Medical Record*, in an article written by A. Vander Veer, M. D., of Albany, N. Y., styled: "Report of Cases of Cholacystotomy for the Treatment of Calculi Lodging in the Common Duct," he recommends, where probing and other methods fail to relieve the obstruction of the common duct from any cause, whether from tumors, stone or other causes, the establishment of a communication between the gall bladder and the bowels, without reference to the common duct. In the article referred to, it is shown clearly that by surgical means, cases of long standing liver disease, whether the gall bladder is distended or not, yet presenting symptoms of billiary obstruction may be relieved by an exploratory operation, the removal of the cause following. These operations show the field of operative surgery widening, and doubtless will lead to many seeking relief from their chronic liver diseases by other means more direct and effectual in many instances than tormenting the stomach and injuring the system with the innumerable kinds of medicines, both patented and otherwise, and for diseases of the liver.

One other reason may be derived from the history of the case under consideration. The cystic duct was not probed, yet complete relief followed. Where, in such a case, distension of the gall cyst exists, and the strength of the patient precludes a very extended examination, by probing or other means, prolong the operation, and thereby increasing the danger, it would be safe to wait after cutting into the gall bladder, for reaction, and further developments, continuing the more radical means whenever found absolutely indicated, and the patient's strength will permit.

